# ^19^F−{^1^H} NMR spectroscopy in weakly orienting solvents for the enantiomeric resolution of fluorinated chiral drugs: The case of fluoxetine

**DOI:** 10.1016/j.jpha.2025.101469

**Published:** 2025-10-10

**Authors:** Vincent Chiapolino, François-Marie Moussallieh, Philippe Lesot, Boris Gouilleux

**Affiliations:** a University of Paris-Saclay, Institute of Molecular Chemistry and Materials of Orsay (ICMMO), Orsay, 91400, France; b National Center for Scientific Research (CNRS), Paris, 75016, France

**Keywords:** Enantiomeric analysis, ^19^F nuclear magnetic resonance, Lyotropic liquid crystal, Poly-γ-benzyl-L-glutamate, Chiral drugs, Fluoxetine, Active pharmaceutical ingredient, Fluorinated drugs

## Abstract

A rapid and simple method combining ^19^F−{^1^H} nuclear magnetic resonance (NMR) and weakly orienting chiral solvents is proposed for the spectral discrimination and accurate quantitation of fluoxetine (FLX) enantiomers. FLX is a well-known bioactive chiral drug in pharmacology with well-established anti-depressant properties (Prozac or Sarafem) and currently used as a racemate. Since 1991, it has been established that the (*S*)-form of FLX shows different pharmacokinetic and pharmacodynamic profiles in comparison to the (*R*)-form, hence the development of novel enantioresolved NMR methods for analyzing FLX is of interest. The reported approach, relying on a commercial polymer, poly-γ-benzyl-L-glutamate (PBLG) as chiral selector, addresses the enantiomeric analysis of FLX hydrochloride solutions with a high level of accuracy in a short time. The influence of experimental parameters, such as solute concentration and temperature on the enantiomeric resolution, is investigated and discussed in depth. In particular, the uniformity and stability of PBLG-based lyotropic liquid crystals (LLCs) in presence of a hydrochloride analyte is assessed for the first time by ^19^F NMR imaging. All the outcomes obtained highlight the analytical potential of this NMR approach for the enantiomeric analysis of fluorinated chiral drugs while the spectral data recorded at various conditions in temperature and mesophase composition provide new valuable insights toward a better understanding of chiral recognition processes in polypeptide orienting media.

## Highlights

• Enantiomeric analysis of hydrochloride fluoxetine is addressed by ^19^F NMR spectroscopy in a chiral orienting solvent.
• The proposed method is fast, relies on a commercial polymer (PBLG), and is suitable with a non-deuterated solvent.
• The achievable chiral resolution allows the presented method to be applied on routine NMR devices.
• The uniformity and stability of a liquid-crystal hosting a drug as a hydrochloride salt is established by ^19^F NMR imaging.
• The influence of solute concentration and temperature on the enantiomeric resolution is investigated.

## Graphical abstract

**Figure:**
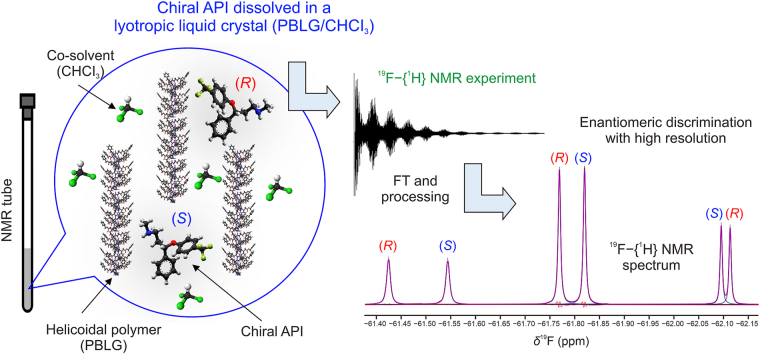

## 1 Introduction

Molecular chirality plays a pivotal role in pharmaceutical chemistry, with over 50% of the drugs currently on the market containing at least one stereogenic center [1,2]. While one enantiomer of a chiral active pharmaceutical ingredient (API) leads to the desired activity on the biological target (eutomer), the other may induce disparate effects, ranging from no activity to opposite or even toxic side-effects (distomer) [3]. Consequently, an increasing number of innovative chiral drugs are being marketed as pure enantiomeric compounds, while some drugs initially used as a racemate are being replaced by the identified eutomer (i.e., chiral switch) to improve the therapeutic efficacy [4,5]. This involves the continued development of rapid, versatile, and complementary analytical tools to determine the enantiomeric purity of a bioactive substance at various stages, from drug synthesis steps to the control of the final commercial medicines [6].

The main analytical methods currently available for the chiral analysis include chiroptical spectroscopy, chiral gas or liquid chromatographies, chiral capillary electrophoresis, chiral mass spectrometry, or liquid-state nuclear magnetic resonance (NMR) in presence of a chiral agent (CA) [7,8]. These CAs can be, on one hand, chiral derivatization agents (CDAs) such as Mosher acid leading to diastereoisomers by covalent bonding and [9], and on the other hand, chiral metal complexes, lanthanide shift reagents, chiral solvating agents (CSAs) known to form diastereoisomeric adducts by means of intermolecular interactions [[10], [11], [12], [13], [14], [15]]. The conversion of enantiomers into diastereomeric entities allows generally the NMR spectral discrimination of enantiomers by variations in their isotropic chemical shifts or those of the CA. Most of the developed CAs offer effective enantiomeric discrimination for only one class of compounds, such as carboxylic acids [16,17], amines [13,[18], [19], [20], [21]], and aliphatic alcohols [9], although cyclodextrins [22] or the recently reported benzamide-based CSAs [23] may be suitable for a wider range of chiral analytes. In addition, a significant number of CSAs reported in the literature have been specifically synthesized and are not commercially available at this time [24]. For them, the users must therefore carry out their synthesis and purification prior to be exploitable in chiral analysis, hampering somewhat the application of this methodology in routine.

Interestingly, the concept of enantiospecific non-covalent interactions between stereoisomers and a CA in isotropic solvents can be extended to orienting media such as chiral lyotropic liquid-crystalline phases based on an enantiopure helical polymer (i.e., the chiral selector) dissolved in routine organic solvents (polar or weakly polar) in appropriate proportions [25,26], or based on smaller non-polymeric entities undergoing a self-assembling process [27,28]. In such chiral weakly orienting media, the chiral analyte adopts an average orientational order that varies from one enantiomer to another [[29], [30], [31], [32]]. This divergence in the molecular orientation results in a difference of three major residual anisotropic NMR interactions, namely residual chemical shift anisotropy (RCSA), residual dipolar coupling (RDC), and residual quadrupolar coupling (RQC), which is at the base of the enantiomeric discrimination in chiral anisotropic media [25,26].

**Fig. 1:**
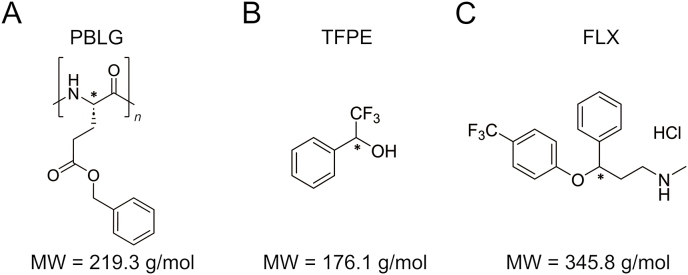
Molecular structure of (A) poly-γ-benzyl-L-glutamate (PBLG), (B) 2,2,2-trifluoro-1-phenylethan-1-ol (TFPE), and (C) fluoxetine (FLX) hydrochloride. The molecular weights (MWs) of PBLG monomer, TFPE, and FLX are given below. The asterisks indicate the stereogenic centers.

In the frame of the enantiomeric analysis of chiral fluorinated APIs (^19^F-APIs), we pioneered in 2024 a promising approach involving anisotropic ^19^F one-dimensional (1D) NMR and a chiral lyotropic liquid crystal (LLC) based on poly-γ-benzyl-L-glutamate (PBLG) [33]. The molecular structure of the PBLG is shown in Fig. 1A. First experimental results obtained with flurbiprofen and efavirenz demonstrated the applicability of the anisotropic NMR approach to chiral APIs involving diverse chemical features, such as carboxylic acid, cyclic carbamate, and aromatic and acetylenic groups. This versatility is also extended to the case of alcohols as presented here with the enantiomeric resolution of 2,2,2-trifluoro-1-phenylethan-1-ol (TFPE) shown in Fig. 1B.

To further highlight the relevance of this methodology in the frame of chiral APIs, we have explored the case of fluoxetine (FLX) (Fig. 1C), a selective serotonin reuptake inhibitor mainly used for the treatment of major depression and obsessive-compulsive disorder [34,35]. The (*S*)-enantiomer of FLX has a longer duration of action and the (*S*)-form of the active metabolite (norfluoxetine) has a superior potency as serotonin inhibitor than its (*R*)-counterpart [36,37]. In addition to the importance of proposing alternative methods for the chiral analysis of this widespread drug, FLX offers two interesting chemical features that may challenge the herein approach. First, this compound includes an amine function which can cause detrimental line-broadening on the NMR spectrum when dealing with polypeptide-based LLCs prepared with apolar solvents (i.e., chloroform and dichloromethane) as previously reported [38]. Second, FLX is usually marketed in a hydrochloride form to achieve desirable formulation properties, such as a greater solubility in water. Dissolution of hydrochloride salts in polypeptide-based LLCs has been very rarely described to date, and hence the potential impact of HCl salts on the stability and uniformity of the liquid-crystalline phase deserves to be investigated.

In this article, we present an exhaustive study on the spectral discrimination properties and quantitation of FLX enantiomers by the combination of ^19^F−{^1^H} 1D NMR and a PBLG/chloroform-based LLC, examining various key experimental parameters (temperature and solute amount). To illustrate the analytical value of this approach, all ^19^F spectral data under discussion have been obtained: i) by the means of routine 1D ^19^F NMR experiments within short acquisition times of no more than a few minutes; ii) after a relatively simple sample preparation involving a commercially available polymer without the need of further purification and with the possibility to use non-deuterated solvents; and iii) by using only a routine medium-field NMR instrument (300 MHz spectrometer, i.e.*,* 7.05 T, and classical NMR probe), demonstrating the suitability of this approach on an NMR equipment representative of common (academic or pharmaceutical) analytical platforms. The ability of yielding uniform and stable mesophases in presence of hydrochloride and amine-contained molecules will be also investigated by ^19^F NMR imaging. The complex role of the solute concentration and temperature onto the resulting enantiomeric resolution is explored in details to provide the best experimental conditions. Finally, the quantitative performance of the enantiopurity determination of APIs using this enantioresolved method will be evaluated in terms of trueness and precision.

## 2 Materials and methods

### 2.1 Chemicals

(*Rac*)-FLX and (*S*)-FLX were purchased from Tokyo Chemical Industry Co., Ltd. (Tokyo, Japan) and Biosynth (Staad, Switzerland), respectively, both with a purity of 98%. TFPE was purchased at Acros Organics (Geel, Belgium). The PBLG polymer was synthetized by the synthesis platform of the Institute of Molecular Chemistry and Materials of Orsay (ICMMO) (Orsay, France). The degree of polymerization (DP) of the PBLG used in this study was estimated at 641 by ^1^H diffusion ordered spectroscopy (^1^H DOSY) NMR. Note here that PBLG is also commercially available (for instance at Merck or Sigma-Aldrich). Chloroform (Carlo Erba, Cornaredo, Italy) used to prepare the liquid-crystalline phases was stabilized with ethanol (0.6%−1.0 %) and dried on molecular sieves. All compounds listed above were used without any further purification.

### 2.2 Sample preparation

The oriented samples were prepared by dissolving an amount of FLX (<30 mg) into dry CHCl_3_ in a flat vial, then the solution was added into a 5-mm NMR tube containing nearby 100 mg of PBLG. The exact amount of chloroform (about 600 mg) was adjusted so that the percentage of total weight of polymer (i.e., mass of PBLG/total mass of sample components) was maintained at 14%, leading to a sample length of approximately 3.5 cm. The exact compositions of each sample are given in Tables S1 and S2. The NMR tube was fire-sealed to avoid CHCl_3_ evaporation over time and then several low-speed centrifugation cycles (e.g., 500 rpm during 30 s) with inversion of the sample between each cycle were carried out to remove matter gradients (Fig. S1). The centrifugation protocol explored in this work and its impact on the liquid crystal uniformity is discussed in Section 3 (see also in the Supplementary data). Once prepared, the samples were stored in the absence of light and at room temperature until analysis.

### 2.3 ^19^F NMR experiments

All ^19^F NMR experiments were conducted on a 7.05 T ($\nu_{0}$ (^19^F) = 282.4 MHz) Bruker NEO spectrometer equipped with a 5-mm BBFO probe (Bruker Corporation, Billerica, MA, USA). ^19^F−{^1^H} 1D NMR spectra were recorded with a 90° radiofrequency pulse length of 15 μs (power of 6.9 W) and the free induction decay (FID) was sampled during 1 s with a ^1^H composite pulse decoupling (WALTZ-16) (the zgig sequence in the Bruker's TopSpin library). The inter-scan delay was shorter than the usual 5 $\times \;T_{1}$ required in quantitative NMR. This improves the signal-to-noise ratio (SNR) per time unit without inducing a quantitative bias due to similar fluorine longitudinal relaxation, *T*_1_(^19^F) for an enantiomeric pair (see Section 3).

Given their short experimental duration (e.g., a few minutes), ^19^F−{^1^H} spectra are not impacted by the natural drift of the magnetic field B_0_, and hence all experiments can be carried out without deuterium lock. The samples can be therefore prepared without deuterated chloroform and without generating any undesired solvent signals on the ^19^F NMR spectra. Under this condition, the shimming process is achieved via an automatic shimming procedure applied on the ^1^H signal of CHCl_3_ to optimize the signal line-shape. Note that the achievement of this last step highly depends on the spatial uniformity of the liquid-crystalline phase [39]. The FIDs were processed using the Mnova software 14.2 (Mestrelab, Santiago de Compostela, Spain), including zero-filling (128 k datapoints), Fourier transform, manual phasing ,and a polynomial (order 5) baseline correction.

The signal integration for the measurement of enantiomeric excess (*ee*%) was achieved by a line-fitting procedure to minimize area biases in cases of enantiomeric signals non-fully baseline separated. This data-processing fits the NMR peaks to a linear combination of Lorentzian and Gaussian functions and finely tunes the height, the linewidth, and the Lorentzian/Gaussian ratio. The linewidth and Lorentzian/Gaussian ratio were not imposed constant for all components of a same ^19^F triplet, in order to take account of any asymmetry (even slight) due to imperfect mesophase uniformity or differential line broadening effects (i.e., cross-correlated relaxation). The *T*_1_(^19^F) values of FLX enantiomers were measured by standard inversion-recovery experiments carried out with 16 incremented inversion delays. 16 scans per increment were accumulated with a repetition time of 10.5 s ensuring a full longitudinal relaxation between scans. The pseudo-2D data were processed by the *T*_1_/*T*_2_ dynamic module of TopSpin 4.0 (Bruker Corporation), where a standard mono-exponential function was used for non-linear fitting.

The *Z*-imaging ^19^F NMR experiments were carried out by 17 successive slice-selective experiments along *Z*-axis (i.e., along the NMR tube length) with a thickness of 0.1 cm each. These slice selections are obtained by a concurrent application of a pulse-field-gradient (22.7 G/cm) and a shaped-pulse (“qsneeze”) of 0.8 ms inducing an excitation bandwidth of 10 kHz. The slice position from the bottom to the top of the sensitive volume of the NMR probe was incremented via the shaped-pulse offset frequency ranging from −80 to +80 kHz.

## 3 Results and discussion

### 3.1 Enantiomeric discrimination by anisotropic ^19^F NMR observables

Within an achiral isotropic medium, the ^1^H-decoupled ^19^F NMR spectrum of molecules containing a CF_3_ group is reduced to a single resonance centered on the isotropic ^19^F chemical shift, *δ*_iso_(^19^F) associated to three homotopic fluorine nuclei (A_3_ spin system). The same mixture dissolved in the PBLG/CHCl_3_ phase leads to a more complex spectral pattern where the CF_3_ group leads to a (1:2:1) triplet centered on the anisotropic ^19^F chemical shift, *δ*_aniso_(^19^F). The successive lines of the triplet are separated by the ^19^F−^19^F total spin-spin coupling constant, $T_{\text{FF}}$ (observed in absolute value), which is equal to three times the residual ^19^F−^19^F dipolar coupling, $D_{\text{FF}}$, between the equivalent fluorine nuclei:

Eq. (1) $$T_{\text{FF}}\left(R\;\text{or}\;S\right)\;=\;3D_{\text{FF}}\left(R\;\text{or}\;S\right)$$

**Fig. 2:**
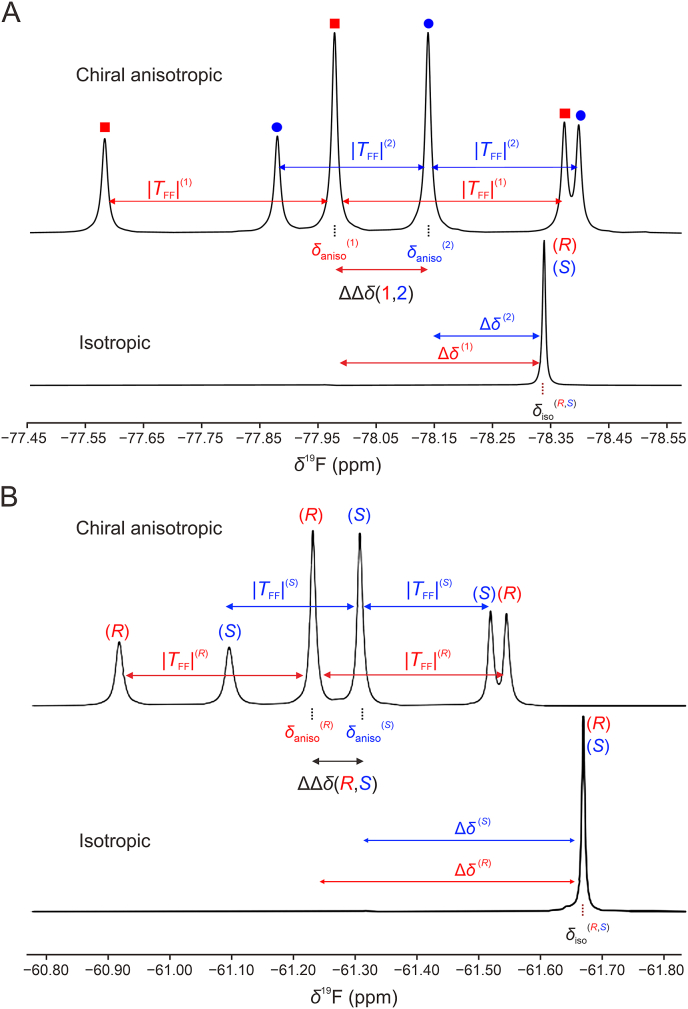
The 282.4 MHz ^19^F−{^1^H} nuclear magnetic resonance (NMR) spectra of (A) (rac)-2,2,2-trifluoro-1-phenylethan-1-ol (TFPE) and (B) (rac)-fluoxetine (FLX) hydrochloride recorded at 26.9 °C in chiral anisotropic poly-γ-benzyl-L-glutamate (PBLG)/CHCl_3_ environment (top) and isotropic CHCl_3_ solution (bottom). The $T_{\text{FF}}$^(1)^ and $T_{\text{FF}}$^(2)^ measured (absolute values) are equal to 111.8 and 73.3 Hz, respectively. The Δ*δ*^(1)^ and Δ*δ*^(2)^ variations are 0.361 and 0.200 ppm, respectively. The *R*/*S* assignment of enantiomers in the FLX racemic mixture was achieved by comparison with a scalemic sample of FLX. The $T_{\text{FF}}$^(*R*)^ and $T_{\text{FF}}$^(*S*)^ measured (absolute values) are equal to 55.4 and 89.5 Hz, respectively. The Δ*δ*^(*R*)^ and Δ*δ*^(*S*)^ variations are 0.042 and 0.131 ppm, respectively.

When chiral recognition occurs between enantiomers, a pair of non-superimposable triplets centered on *δ*_aniso_(*S*) and *δ*_aniso_(*R*) are expected, one corresponding to one enantiomer. Spectral enantiomeric discrimination is therefore achieved through differences of both residual ^19^F chemical shift anisotropy ^19^F-RCSA and residual ^19^F−^19^F dipolar coupling (^19^F−^19^F)-RDC. This concept is well illustrated in the case of TFPE (Fig. 2A) and FLX (Fig. 2B), two compounds involving various functional groups. The (*R*)- and (*S*)-enantiomers assignment can be achieved by comparison with scalemic series involving a small *ee*% (approximately 10%), such as in the case of FLX (Fig. 2B). In this ^19^F−{^1^H} spectrum, recorded at 300 K with a lyotropic liquid-crystalline phase containing 3.3 mg (i.e., 23.8 mmol/L) of FLX dissolved in PBLG/CHCl_3_ with a 14% mass ratio in polymer, the variations ΔΔδ(R,S) and $\left|T_{\text{FF}}\left(R\right)\right|-\left|T_{\text{FF}}\left(S\right)\right|$ are equal to 0.09 ppm and 34.1 Hz, respectively. Given the spectrometer magnetic field (B_0_ = 7.05 T, $\nu_{0}$ (^19^F) = 282.4 MHz), ΔΔδ(R,S) corresponds here to 25.4 Hz, so that the (^19^F–^19^F)-RDC is here the key NMR observable in the spectral enantiomeric discrimination of FLX.

The underlying mechanisms governing chiral recognition in polymer-based LLCs are not yet fully understood, and deciphering them remains an on-going challenge, as evidenced by recent theoretical work based on molecular dynamics simulations [40]. We have explored the FLX-PBLG interactions to get some insights on the spatial arrangement of this API at the vicinity of PBLG side chains and to track potential discrepancies between enantiomers explaining the chiral discrimination. This study, based on saturation transfer difference (STD) NMR experiments [[41], [42], [43]], is presented in detail in the Supplementary data. In brief, we can outline an arrangement of FLX in contact with PBLG, in which aromatic groups of FLX interact with the terminal phenyl of PBLG while the amino alkyl chain of the API is located close to the carbonyl part of the polymer (Fig. S2). The asymmetric center of FLX is at similar distance from the ester and aromatic groups of PBLG. The same trend is observed for both enantiomers with only slight differences, suggesting a similar arrangement of FLX enantiomers in contact with the PBLG side chains. In spite of these minor variations, the enantiomeric resolution of FLX is well observed as seen in Fig. 2B.

Returning to the anisotropic FLX spectrum, it is worth noting the significant discrepancies in the relative peak intensities observed between the central and external components of triplets compared to the expected (1:2:1) ratio. The linewidths measured at half maximum are clearly different for the deshielded, central, and the shielded components of the triplet, revealing a differential line-broadening within a same triplet. This feature is not related to an inappropriate magnet shimming process or partially inhomogeneous samples as this feature remains regardless the spectrometer used and the operator involved. This differential line-broadening is complex and probably arises from interferences of relaxation processes that can occur in case of nuclei involving a strong shielding anisotropy, such as ^19^F, ^31^P, or ^15^N [44]. Previous observations of differential line-broadening have been also reported in liquid-state NMR considering fluorinated-aromatic compounds with various spin-systems (e.g., AMX and AX_2_) due to cross-correlation between dipole-dipole and dipole-CSA relaxation mechanisms [45,46].

To investigate the potential cross-correlation relaxation in the present case, inversion-recovery experiments have been carried out to extract the longitudinal relaxation times *T*_1_(^19^F) for each component of a given triplet. The values measured vary from one line to another within the same triplet that is consistent with cross-correlated relaxation (Table 1). In the herein chiral environment, this cross-correlation effect is similar for the both enantiomers, so it cannot be used for (*R*)- and (*S*)-stereoisomers assignment. In the same vein, the *T*_1_(*R*) and *T*_1_(*S*) show no differences (or at least within the uncertainty of the measurement) in longitudinal relaxation of the enantiomers (Table 1 and Fig. S3).

**Table 1:** Longitudinal relaxation times *T*_1_(^19^F) measured peak by peak for the two triplets of fluoxetine (FLX) hydrochloride enantiomers. Results obtained on a sample with 20 mg of (rac)-FLX in poly-γ-benzyl-L-glutamate (PBLG)/CHCl_3_ at 26.9 °C.

*δ*^19^F (ppm)	Component^a^ (d, c, s)	Assignment (*R*, *S*)	*T*_1_(^19^F)^b^ (ms)	RSD^c^ (%)
−60.77	d	*R*	886.9 ± 26.2	1.5
−61.17	c	*R*	1070.0 ± 56.4	2.6
−61.56	s	*R*	1005.5 ± 37.3	1.9
−60.83	d	*S*	885.7 ± 30.8	1.7
−61.19	c	*S*	1067.4 ± 67.4	3.2
−61.55	s	*S*	981.6 ± 57.5	2.9

a d, c, s: deshielded, central, and shielded resonance of the triplet.

b Mean *T*_1_(^19^F) value from five successive experiments, the uncertainty corresponds to the 95% confidence interval (95%CI) determined here as two times the standard deviation (SD).

c Relative standard deviation (RSD) computed on the same five successive experiments.

### 3.2 Spatial uniformity and stability of LLCs

LLCs based on polypeptides such as the PBLG polymer with a positive molecular magnetic susceptibility anisotropy (${\Delta \chi }_{m}$ > 0) lead to cholesteric mesophases that are transformed almost instantaneously into chiral nematic media with director, $\vec{n}$, parallel to B_0_ under the effect of an intense magnetic field [47]. The spatial uniformity of such mesophases is of primary importance on the quality of the resulting NMR spectra. As studied by Gil and co-workers [39], inhomogeneities cause a variation of the orientational ordering of the guest molecules (solute and co-solvent) according to their spatial position in the sample. In such case, the residual anisotropic NMR interactions become spatially dependent leading to severe line-broadening and strong signal distortions of resonances along with possible complications for the shimming process.

The characteristics (uniformity and stability) of the PBLG liquid-crystalline phases in presence of HCl salts as solute are not well-known. A previous report from Samulski and co-workers [48] underlined the significant impact of trifluoroacetic acid on the ${\Delta \chi }_{m}$ of PBLG and also on the mesophase fluidity. Thiele and co-workers [49] further explored the influence of various acids and showed the destabilization of PBLG-based liquid crystals by helix-to-random-coil transition with increasing acid content or the acid strength (example of trifluoroacetic acid (TFA)). In this context, to ascertain the quality of the prepared PBLG-based LLCs in presence of hydrochloride salt of FLX, 1D *Z*-imaging ^19^F NMR has been applied at different days since sample preparation in order to characterize the uniformity and stability of the mesophases. This ^19^F *Z*-imaging 1D experiment consists in recording a series of ^19^F−{^1^H} spectra slice by slice along *Z*-axis (i.e., along the NMR tube) (Fig. S4). The resulting data are presented as pseudo-2D spectra (*δ*^19^F, Z) in contour 2D plot with horizontally the ^19^F NMR dimension and vertically the position along *Z*-axis.

**Fig. 3:**
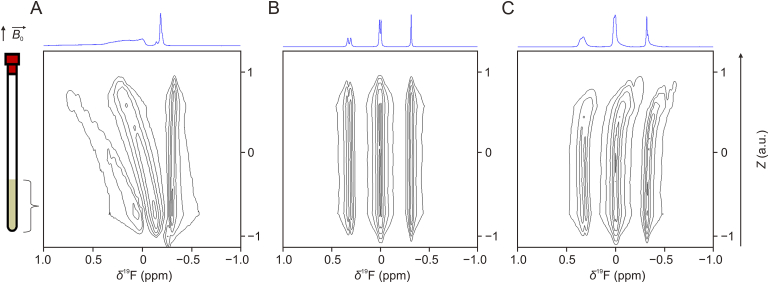
^19^F *Z*-imaging contour plot maps of poly-γ-benzyl-L-glutamate (PBLG)/CHCl_3_ mesophase containing 15 mg of fluoxetine (FLX) hydrochloride recorded the day of the sample preparation: (A) without any homogenization, (B) after 10 cycles of centrifugation procedure (500 rpm, 30 s) with nuclear magnetic resonance (NMR) tube inversion between the successive cycles, and (C) with manual homogenization based only on gravity flow of the lyotropic liquid crystal (LLC) (no centrifugation). The one-dimensional (1D) spectra (in blue) displayed in *F*_2_ projections correspond to the ^19^F−{^1^H} 1D spectra recorded on the entire sensitive volume of the NMR probe. Note the non-Lorentzian shape of the lines due to non-ideal LLC uniformity on Figs. 3A and C.

Immediately after the sample preparation (sealed tube) and without any homogenization procedure, the resulting mesophase is severely inhomogeneous (matter gradients) and the ^19^F−{^1^H} spectra recorded on the whole sensitive volume of the NMR probe are not exploitable (Fig. 3A), even after several days (Fig. S5). Multiple cycles of centrifugation at moderate rotation speed (e.g., 10 times 30 s at 500 rpm) with inversion of the sample between each cycle generally leads to a spatially uniform mesophase. This is shown in Fig. 3B, where parallel straight lines along *Z*-axis and an overall ^19^F−{^1^H} spectrum with suitable line-shape and resolution are obtained. Alternatively, when centrifugation of the NMR tube is not possible (no centrifuge available), manual homogenization based on gravity flow of the mesophase can be envisaged, especially in cases of fluid samples. For FLX samples, flipping the NMR tube up and down several times (e.g., 10 times) before NMR experiments leads also to a uniform mesophase as soon as three days after the sample preparation with comparable results (Fig. S6). Note that at the day of the sample preparation, such a manual homogenization procedure does not produce a sufficient uniformity for a suitable enantiomeric resolution (Fig. 3C). This issue could be, however, overcome by targeting only a uniform portion of the sample using spatially-resolved NMR experiments [50]. This strategy is currently in progress.

Regarding the stability, ^19^F NMR and *Z*-imaging experiments have been performed until 10 days after the sample preparation. No degradation of the mesophase has been observed in this time-scale while the spatial uniformity has been maintained (Fig. S7). To check whether this uniformity is maintained under varying experimental conditions, a second LLC sample with a different FLX concentration (36.1 mmol/L) has been monitored at two different temperatures (27 and 37 °C). The ^19^F *Z*-images clearly show (Fig. S8) uniform LLCs at this concentration and for both temperatures.

In overall, these results demonstrate that the presence of FLX as an HCl salt does not prevent the production of uniform liquid crystals stable over time, which are robust again changes in experimental conditions.

### 3.3 Optimization of the enantiomeric resolution

To improve the reliability of *ee*% determination in the case of FLX, the enantiomeric resolution of anisotropic ^19^F−{^1^H} experiments is mainly optimized as a function of the concentration in solute and sample temperature. These two experimental parameters have recently shown significant effects on the average orientational order adopted by some chiral APIs in a PBLG-based mesophase [33]. Another parameter impacting the magnitude of RCSA and RDC is the concentration in polymer into the organic solution containing a helicogenic solvent (here CHCl_3_) and the analyte. The higher the concentration, the greater the degree of orientation of the mesophase (and so the magnitude of anisotropic NMR observables of analytes) [51], but at the expense of an undesired line-broadening due to higher viscosity of the medium. A good balance lies in a mass ratio of 12%–15% in PBLG, leading to a stable aligning system (without isotropic domain) while reasonable linewidths varying from 1.5 to 5 Hz at half-maximum, are observed. In what follows, all PBLG samples have been prepared with a constant mass ratio of polymer of 14%. The sample compositions are detailed in Tables S1 and S2.

### 3.4 Effect of solute concentration

**Fig. 4:**
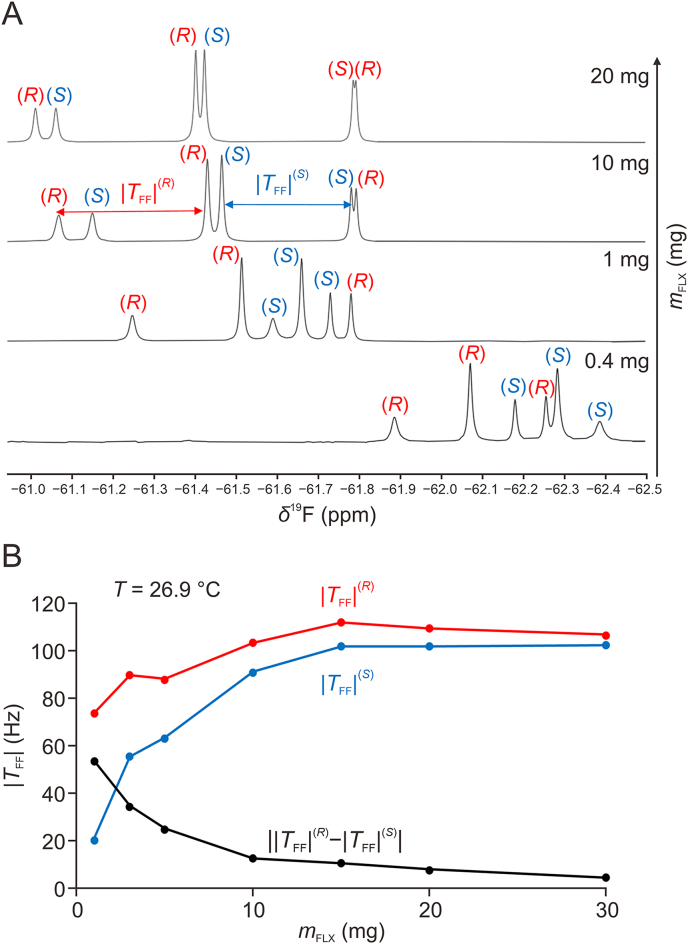
Influence of the mass of fluoxetine (FLX) hydrochloride in the anisotropic ^19^F−{^1^H} nuclear magnetic resonance (NMR) spectra. (A) Series of 282.4 MHz ^19^F−{^1^H} NMR spectra recorded in poly-γ-benzyl-L-glutamate (PBLG)/CHCl_3_ as a function of the FLX: from 20 mg (145 mmol/L) at the top, to 0.4 mg (2.9 mmol/L) at the bottom. All spectra were recorded at 26.9 °C. (B) Experimental variation of the total coupling $\left|T_{\text{FF}}\right|$ for both enantiomers as a function of the FLX content. The black curve shows the variation of absolute values of the difference of $\left|T_{\text{FF}}\right|$ between enantiomers: $\left|\left|T_{\text{FF}}\left(R\right)\right|-\left|T_{\text{FF}}\left(S\right)\right|\right|$.

The effect of concentration of analyte into the PBLG-based LLCs has been examined with an increasing amount of FLX from 1 mg (i.e., 8.0 mmol/L) to 30 mg (i.e., 216.3 mmol/L). As highlighted in Fig. 4A, the solute concentration has here an impact on the measured $\left|T_{\text{FF}}\right|$ according to the concentration range. Slight variations are observed at high concentrations of FLX while the $\left|T_{\text{FF}}\right|$ values significantly drop for a FLX content lower than 10 mg (i.e., 72.2 mmol/L). It must be noted that these variations of residual ^19^F–^19^F dipolar coupling are different from one enantiomer to another (Fig. 4B). It is therefore possible to enhance the enantiomeric resolution by tuning the FLX concentration in the mesophase. This variation of $\left|T_{\text{FF}}\right|$ between enantiomers is more accentuated for samples at lower FLX concentrations. For instance, in the range of 1–10 mg of FLX (i.e., 8.0−72.2 mmol/L), large differences of total coupling constant between enantiomers ($\left|T_{\text{FF}}\left(R\right)\right|-\left|T_{\text{FF}}\left(S\right)\right|$) are observed, up to a 53 Hz difference. As the dipolar coupling interaction does not depend on the strength of the magnetic field B_0_, such a frequency difference could be suitable for the enantiomeric analysis with spectrometers operating at lower magnetic fields.

The difference of anisotropic chemical shift between the two enantiomers |ΔΔδ(R,S)| evolves in a similar manner with respect to the FLX concentration. Slight variations are observed at high concentrations: from |ΔΔδ(R,S)| = 0.02 ppm at 30 mg (i.e., 216.3 mmol/L) to 0.04 ppm at 10 mg (i.e., 72.2 mmol/L) while the anisotropic chemical shift differences between enantiomers increase significantly at lower FLX contents reaching |ΔΔδ(R,S)| = 0.14 ppm for 1 mg of FLX (i.e., 8.0 mmol/L). All the spectral data in terms of coupling values and difference of chemical shifts are listed in Tables S3–S10.

Based on these observations, the concentration range 8.0–72.2 mmol/L leads to a high enantiomeric resolution while the sensitivity remains sufficiently high for quantitative analyses, even with a short experimental duration. The variation of these anisotropic observables with respect to the solute concentration relies on a complex interplay between solute-solute, solute-solvent, and solute-polymer interactions, which is rather difficult to decipher and simply rationale to date. However, it is possible to explore certain hypotheses in an attempt to interpret these results. The first point to be investigated is the impact of the solute on the orienting properties of the liquid-crystalline phase. As previously established, the presence of small quantities of acid (e.g., 2%–10%weight) can significantly disturb the orienting properties of PBLG mesophase (in particular the degree of order) manifested by a drop of the residual ^2^H quadrupolar coupling (^2^H-RQC) observed on the ^2^H signal of CDCl_3_ when used as organic co-solvent [49]. As an hydrochloride salt, FLX may have an impact to the mesophase orienting properties, especially at high content such as 4%weight (i.e., 30 mg of FLX). To address this question, the ^1^H−^13^C spin-spin total coupling of the co-solvent (here CHCl_3_) defined as $T_{\text{CH}}=J_{\text{CH}}+2D_{\text{CH}}$, directly measured on the ^1^H NMR spectra (signal of ^13^C-satellites of the solvent), has been tracked as a function of the FLX concentration (Fig. S9). As seen, the absolute values of this anisotropic observable remain quite constant on the FLX-concentration range explored with a relative standard deviation (RSD) of 0.76% (Fig. S10 and Table S11), thus excluding a significant modification of the mesophase orienting properties through FLX adding.

The variation of the anisotropic observables of FLX with respect to its concentration is thus intrinsic to the solute, and hence the decrease in $D_{\text{FF}}$ should be discussed from the analysis of Eq. (2):

Eq. (2) $$D_{\text{FF}}\;=\;-k_{\text{FF}}\;\times \;\frac{S_{\text{FF}}}{r_{\text{FF}}^{3}}\;\text{with}\;S_{\text{FF}}\;=\;\left\langle \frac{3\;\cos^{2}\theta_{\text{FF}}^{B_{0}}-1}{2}\right\rangle$$

**Fig. 5:**
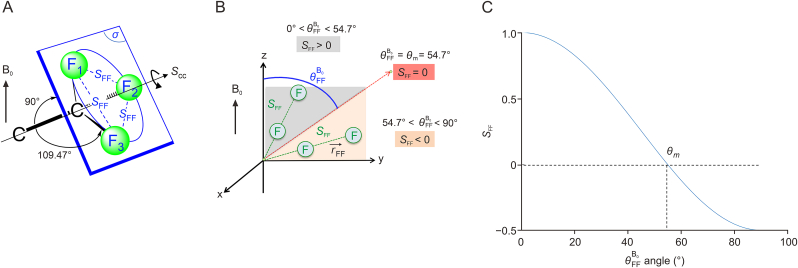
Local order parameters related to a CF_3_ group. (A) Visual representation of the three homotopic fluorine nuclei of the CF_3_ group. (B) Example of relative positions of two F ^….^F vectors with respect to axis B_0_ and leading to positive and negative $S_{\text{FF}}$ values. (C) Variation of the local order parameter $S_{\text{FF}}$, according to the trigonometric term, (3cos^2^$\theta_{\text{FF}}^{B_{0}}$ −1)/2 *versus*$\theta_{\text{FF}}^{B_{0}}$. $\theta_{m}$ is the magic angle (i.e., 54.7°) that cancels out the *S*_FF_ value.

In this equation, *r*_FF_ is the distance between each fluorine atom within the CF_3_ group, $S_{\text{FF}}$ is the local order parameter associated with the internuclear direction between the coupled fluorines, $\theta_{\text{FF}}^{B_{0}}$ is the angle between this internuclear direction and the magnetic field B_0_ axis, and $k_{\text{FF}}$ is a constant related to the dipolar interaction ($k_{\text{FF}}$ = 106.30 kHzÅ^3^) (Figs. 5A and B). The brackets < … > stand for an ensemble average.

Analysis of Eq. (2) indicates that the reduction of the RDC can be due to either a reduction of the generalized degree of order (GDO) experienced by the analyte in average, or a progressive change of the orientation of this molecule as the $\theta_{\text{FF}}^{B_{0}}$ angle approaches the magic angle θ_m_, which in turn cancels the dipolar interaction (Fig. 5C). Under the latter hypothesis, after decreasing continuously to zero, the (^19^F−^19^F)-RDC should then increase (in absolute value) as $\theta_{\text{FF}}^{B_{0}}$ would move away from θ_m_. This investigation has been thus pursued with further diluted samples (i.e., lower than 8.0 mmol/L). The ^19^F−{^1^H} NMR spectra obtained are markedly different from those obtained in the previous concentration range of 8.0–216.3 mmol/L (Fig. 4A). If we now examine the changes in the dipolar coupling over this wide range of concentrations, focusing for example on the (*S*)-isomer, we can observe that $\left|T_{\text{FF}}\left(S\right)\right|$ decreases continuously up to a certain concentration ($\theta_{\text{FF}}^{B_{0}}$ approaching $\theta_{m}$), then increases (in absolute value) at lower concentrations ($\theta_{\text{FF}}^{B_{0}}$ moves away from $\theta_{m}$). This observation is therefore consistent with a progressive reorientation of this enantiomer with respect to the FLX concentration. Note that this variation would involve a sign change of the $T_{\text{FF}}$ value that is not directly visible on the ^19^F−{^1^H} NMR spectra as the coupling constants are measured in absolute value. Yet, a sign change is here consistent with the opposite differential line-broadening observed in the spectrum at the bottom of Fig. 4A compared to those shown at higher concentrations, since the line-broadening/narrowing of the shielded/deshielded components of a signal induced by cross-correlation relaxation depends on the sign of the coupling [44].

### 3.5 Temperature effect

**Fig. 6:**
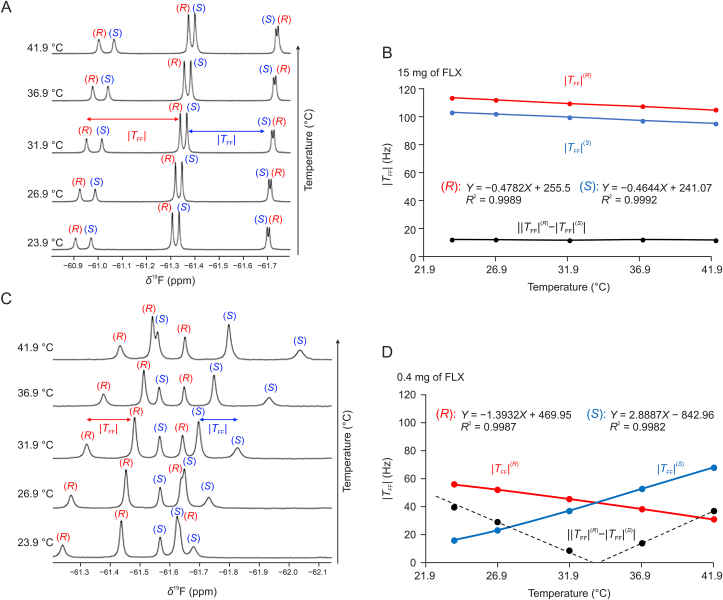
Influence of the temperature (°C) on the ^19^F−{^1^H} nuclear magnetic resonance (NMR) spectra of fluoxetine (FLX) hydrochloride in poly-γ-benzyl-L-glutamate (PBLG)/CHCl_3_. (A, B) Influence of temperature (°C) with 15 mg of FLX: stacked spectra recorded from 23.9 to 41.9 °C (A) and variation of $\left|T_{\mathrm{FF}}\left(S\right)\right|$ and $\left|T_{\mathrm{FF}}\left(R\right)\right|$ values vs. temperature (°C) (B). (C, D) Influence of temperature (°C) with 0.4 mg of FLX: stacked spectra recorded from 23.9 to 41.9 °C (C) and variation of $\left|T_{\mathrm{FF}}\left(S\right)\right|$ and $\left|T_{\mathrm{FF}}\left(R\right)\right|$ values vs. temperature (°C) (D). The black curve corresponds to $\left|\left|T_{\mathrm{FF}}\left(R\right)\right|-\left|T_{\mathrm{FF}}\left(S\right)\right|\right|$.

Another key parameter controlling the orientation properties of the LLC and, subsequently the analyte, is the sample temperature. A new investigation ascertains the influence of temperature (*T*) on the same anisotropic observables has therefore been conducted. In principle, both RCSA and RDC can be used with that respect. However, the anisotropic chemical shift observed in LLCs implies an isotropic and an anisotropic contribution of the shielding tensor, both of which can vary with temperature. This complicates therefore the analysis of the temperature effect on the solute orientation via changes in anisotropic chemical shift. In contrast, dipolar coupling does not contain any isotropic character, making the $T_{\text{FF}}$ coupling constant a much better probe for studying solute orientation under temperature changes. At high concentrations (72.2–216.3 mmol/L), the coupling values $\left|T_{\text{FF}}\right|$ measured for each enantiomer decrease slightly linearly with *T* (Figs. 6A and B). This trend is intuitively expected, since increasing temperature leads to a reduction in the degree of order of the liquid-crystalline phase until a state of isotropy is reached. Since the slope of the linear function for each enantiomer, around 0.5 Hz/°C, is similar (blue and red curves in Fig. 6B), the difference in dipolar coupling between enantiomers is considered as constant over this temperature range of 24–42 °C. This behavior is observed for all FLX concentrations, from 216.3 to 23.8 mmol/L.

Interestingly, in the case of diluted samples (e.g., 0.4 mg of FLX), the effect of temperature is rather unexpected since the variation of $\left|T_{\text{FF}}\right|$ of the CF_3_ group is now enantiospecific (Figs. 6C and D). Indeed, the slope of the $\left|T_{\text{FF}}\right|$ linear variation with *T* is now significantly different (−1.4 vs. 2.9 Hz/°C) from one enantiomer to another (see red and blue plots in Fig. 6D). This implies that the temperature can be adjusted to improve the enantiomeric resolution at concentrations of FLX lower than 8.0 mmol/L. More fundamentally, this unexpected increase of $\left|T_{\text{FF}}\right|$ experienced by the (*S*)-isomer with temperature could be explained by a change of its average orientation in the mesophase influencing the local order parameter $\left\langle S_{FF}\right\rangle$ associated with its CF_3_ group. This phenomenon apparently predominates over the reduction of the global decrease in ordering in this restricted temperature range (24–42 °C). An increase in *T* well above 42 °C would ultimately lead to a decrease in the values of $\left|T_{\text{FF}}\right|$ for both enantiomers as we approach the transition temperature to the isotropic state.

### 3.6 Quantitation of enantiomers in racemic and scalemic series

The determination of the *ee*% of an API by NMR is by essence a quantitative measurement. NMR spectroscopy is a well-established quantitative technique, provided certain conditions are respected during acquisition and processing step. Therefore, mastering the potential quantitative biases deserves attention in the sake of accurate measurement of the *ee*%. The most important one is the uncompleted longitudinal relaxation during the repetition time (*t*_R_) between two successive scans, at various extent between nuclei with different characteristic relaxation times *T*_1_. In quantitative NMR experiments, this bias is usually circumvented with a long *t*_R_ fixed at five times the longest *T*_1_ of nuclei to be probed. However, this quantitative condition is yielded at the expense of SNR per time unit.

**Fig. 7:**
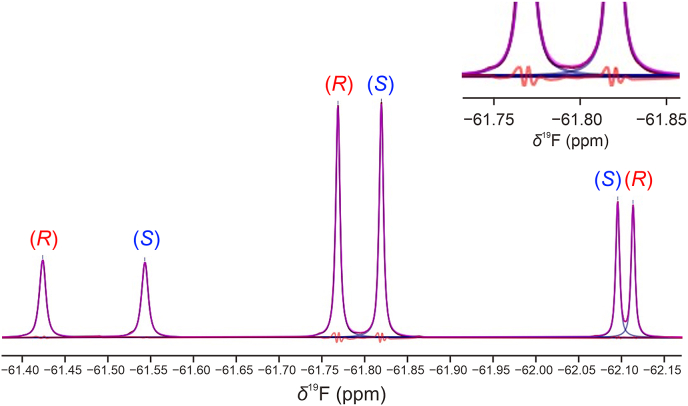
Superimposed experimental (brown) and reconstructed (pink) ^19^F−{^1^H} spectra after line-fitting treatment. The inset shows a vertical zoom of the two central lines of the fluoxetine (FLX) signals with the residue curve (red) between the experimental and reconstructed spectra. The spectrum was recorded on 10 mg of (rac)*-*FLX hydrochloride dissolved in poly-γ-benzyl-L-glutamate (PBLG)/CHCl_3_ at 26.9 °C, and enantiomeric excess (*ee*%) was calculated using the peak areas of two most deshielded lines (i.e., left components of triplets) of (*R*)- and (*S*)-isomers.

This annoying but necessary requirement can be alleviated in the case of a relative quantification of enantiomers because no significant differences of relaxation times *T*_1_ between enantiomers have been so far reported (also in weakly aligning LLCs), at the best of our knowledge. This is again verified here in the case of FLX where no differences of *T*_1_(^19^F) (greater than the measurement uncertainty) have been observed between the two enantiomers. This occurrence allows to record ^19^F−{^1^H} 1D NMR spectra in partial saturation conditions without inducing a systematic quantitative bias. A significant improvement in the SNR per time unit is therefore gained, thus enhancing the repeatability (precision) and detection limit of the method for a given experimental duration, *t*_exp_. ^19^F−{^1^H} 1D NMR experiments (*t*_exp._ ≈ 4 min) were performed on both racemic and scalemic series and the experimental *ee*% determined as follows:

Eq. (3) $$ee\left(i\right)\%\;=\;100\;\times \;\frac{A_{i}\;-\;A_{j}}{A_{i}\;+\;A_{j}}$$

where the subscript “i” is related to the enantiomer in excess (major isomer) and A is the signal area. In practice, the signal integration is performed by a line-fitting procedure (Fig. 7) where the experimental lines are reconstructed by a linear combination of Lorentzian and Gaussian functions.

The trueness of the method, i.e., deviation between the expected and experimental values of the measurement, was evaluated only on the racemic sample as the expected *ee* = 0% does not suffer from weighting uncertainties during the sample preparation. In practice, the deshielded components of the two triplets were selected for this purpose because they provide the best enantiomeric resolution (but not the better SNR). The precision (here considered as the short-term repeatability) is determined by the standard deviation (SD%) of the measurement calculated on a series of five successive identical NMR experiments.

The trueness determined on the racemic mixture is of 0.09% (Table 2, row 1), which highlights the excellent agreement between theoretical and experimental *ee*% values and the absence of quantitative biases. This high level of accuracy is achieved without any prior calibration procedure, reference materials, or post-correction factors.

**Table 2:** Enantiomeric excess (*ee*%) measured on racemic and scalemic fluoxetine (FLX) hydrochloride samples in poly-γ-benzyl-L-glutamate (PBLG)/CHCl_3_.

*ee*_theo_ (*S*) (%)	*ee*_exp_ (*S*)^c^ (%)	Deviation (%)	SD^d^ (%)	SNR (*R*)^e^	SNR (*S*)^e^
0^a^	0.09	0.09	0.08	960	931
8.94 ± 1.14^b^	8.87	0.07	0.43	1524	1791
29.9 ± 1.14^a^	28.1	1.84	0.49	444	734

a Total mass of FLX of 10 mg.

b Total mass of FLX of 15 mg.

c The experimental *ee*% corresponds to the mean values calculated on five successive nuclear magnetic resonance (NMR) experiments of 4 min.

d Standard deviation (SD) value on five successive experiments.

e Signal-to-noise ratio (SNR) measured on the deshielded component of the triplet.

For an experimental duration of 4 min at 282.4 MHz, the SNR obtained on the signals of FLX enantiomers is of the order of 1800 (for the central lines of the triplets) and about 950 for the deshielded components at a concentration of 36 mmol/L per enantiomer (see row 1 of Table 2). This ensures an excellent precision (SD = 0.08%) for this 10 mg FLX sample, which enables here the measurement with an uncertainty of ± 0.16%, if a 95% confidence interval (95% CI) is considered (calculated as 2 × SD (%)). Interestingly, the SD (%) value does not vary significantly with the total mass of FLX for scalemic samples, as seen in Table 2, by comparing entry 2 (15 mg of FLX) and entry 3 (10 mg of FLX). Despite a difference of SNR for these two samples, the precision remains similar (SD of 0.43% vs. 0.49%). This suggests that the precision of the *ee%* measurement is here dominated by the repeatability of the line-fitting procedure, rather than the sensitivity of the NMR experiment.

## 4 Conclusion

Enantiomers of FLX are spectrally discriminable and quantifiable by ^19^F NMR performed in a weakly-orienting chiral solvent. The determination of *ee*% can be achieved with a high level of accuracy and in a rapid NMR experiment (e.g., about 4 min). The reported method is applied without the support of any a prior calibration procedure or correction factor, while the polymer used to generate the liquid-crystalline phase is commercially available. Interestingly, this work underlines the possibility of generating uniform and stable liquid-crystalline phases in case of hydrochloride molecules. The oriented samples are sufficiently uniform after several cycles of centrifugation to provide ^19^F NMR spectra with a suitable enantiomeric resolution at the day of the sample preparation. In the absence of a centrifuge, manual homogenization can be considered with comparable results, but only from three days after sample preparation.

Based on the presented ^19^F NMR results, the range 1–10 mg (i.e., 8.0−72.2 mmol/L) of FLX hydrochloride (molecular weight = 345.8 g/mol) is optimal in terms of enantioresolution and sensitivity for few minutes of NMR experimentation. In this range, the difference of ^19^F−^19^F total coupling between enantiomers can reach 53 Hz at 26.9 °C, which offers a comfortable enantiomeric resolution even with routine or even low-field benchtop NMR spectrometers as the magnitude of the dipolar coupling interaction is independent of the magnetic field strength.

From a practical aspect, chiral anisotropic ^19^F−{^1^H} NMR applied to fluorinated APIs has several advantages: i) recording 100% abundant nuclei with a high gyromagnetic ratio, ii) no heteronuclear couplings increasing the distribution of peaks, iii) simple spectral pattern to analyze (e.g., a singlet for an isolated fluorine nucleus or a triplet for a CF_3_ group), and iv) the absence of undesired signals due to the presence of non-fluorinated impurities or traces of water in the co-solvent or polymer.

From a more fundamental point of view, this work also shows the significant influence of the FLX concentration on the magnitude of the (^19^F−^19^F)-RDC in an enantiospecific manner. This parameter is therefore important for the achievable enantiomeric resolution. These variations of dipolar coupling can be regarded as a progressive change of the average orientation of FLX enantiomers according to their concentration in the chiral mesophase. The sample temperature also influences the magnitude of this coupling but in a different way with respect to the concentration range: a similar decrease of the (^19^F−^19^F)-RDC for both (*R*)- and (*S*)-FLX is observed from 23.8 to 216.3 mmol/L, while the temperature effect varies from one enantiomer to another in more diluted samples. The origins of such changes of enantiomer average orientations arise from a complex interplay between solute-polymer and solute-solute interactions that are both sensitive to temperature and solute concentration. Deciphering these complex mechanisms underlying the chiral recognition in such liquid oriented media remains an on-going challenge, for which these reported outcomes are insightful materials for a better understanding.

## CRediT authorship contribution statement

**Vincent Chiapolino:** Writing – review & editing, Investigation, Formal analysis, Data curation. **François-Marie Moussallieh:** Writing – review & editing. **Philippe Lesot:** Writing – review & editing, Validation, Supervision, Formal analysis. **Boris Gouilleux:** Writing – review & editing, Writing – original draft, Validation, Supervision, Project administration, Methodology, Funding acquisition, Formal analysis.

## Declaration of competing interest

The authors declare that they have no known competing financial interests or personal relationships that could have appeared to influence the work reported in this paper.
