# Supplementary material for: 19F−{1H} NMR spectroscopy in weakly orienting solvents for the enantiomeric resolution of fluorinated chiral drugs: The case of fluoxetine
Source: J Pharm Anal. 2025 Oct 10;16(5):101469. doi: 10.1016/j.jpha.2025.101469 (PMC13193786; doi:10.1016/j.jpha.2025.101469)
Supplement: Multimedia component 1 [file mmc1.docx]

**^19^F-{^1^H} NMR Spectroscopy in Weakly Orienting Solvents for the Enantiomeric Resolution of Fluorinated Chiral Drugs: The Case of Fluoxetine**

**Table of content**

**I. Chemicals and lyotropic liquid crystals**

**II. Sample composition of oriented samples**

**III. Investigation of Fluoxetine-PBLG contacts**

**IV. Longitudinal relaxation behavior of Fluoxetine**

**V. Spatial uniformity and stability of the lyotropic mesophases**

**VI. NMR spectral data of (*rac*)-Fluoxetine dissolved in PBLG/CHCl_3_ at various concentrations and temperatures**

# I. Chemicals and lyotropic liquid crystals

**Chemicals.** (*Rac)*-Fluoxetine ((*rac)*-FLX) was purchased at Tokyo Chemical Industry (TCI) with a purity of 98%, (*S*)-FLX was purchased at Biosynth with a purity of 98%. Poly-γ-benzyl-L-glutamate (PBLG) was synthetized by the synthesis platform within the Institut de Chimie des Molécules et des Matériaux d’Orsay (ICMMO, UMR CNRS 8182). The degree of polymerization (DP) of this PBLG was estimated at 641 by ^1^H diffusion ordered spectroscopy (DOSY) NMR. Yet, this type of polymer is also commercially available (for instance at Merck). Protonated Chloroform used to prepare the liquid-crystalline phases was analytical grade with ethanol as stabilizer (< 0.004%) and dried by molecular sieves. All compounds listed above were used with any further purification.

**NMR sample preparation.** Around 100 mg of PBLG was weighted and added to a 5-mm NMR tube. The chiral solute to be studied was weighted and dissolved in CHCl_3_ (around 600 mg) in a flat vial. This solution was then transferred to the NMR tube and chloroform was here added if necessary - so that the total wt% of PBLG (*i.e.*, m_PBLG_ / m_tot_) is maintained at around 14%. The NMR tube is then flame sealed to avoid CHCl_3_ evaporation and several low-speed centrifugation cycles of the tube at short time repetition (*e.g.* 500 rpm during 30 s) are carried out to limit solute orientation inhomogeneities (mainly due to matter gradients). The NMR tube is inverted between each centrifugation cycle. The centrifuge used is a ROTOFIX 46 H purchased from Hettich Lab Technology, Germany (see **Fig. S1A**). In the case of manual homogenization, the NMR tube was inverted ten times, each time allowing the liquid-crystalline phase to naturally flow out completely by gravity. The birefringence of mono- or bimesophasic samples is assessed using a homemade polarizer where NMR tubes are located between two crossed polarization filters (see **Fig. S1B**).

B

**Fig. S1:** (A) The ROTOFIX 46 H centrifuge used to prepare the oriented samples. Note the *homemade* *NMR tube holder to protect the NMR sample and avoid its breaking during high-speed rotations. Here the 5-mm NMR tubes (head up) are sealed. (B) Picture of a homemade polarizer built to assess the birefringence of oriented samples, outside the magnetic field.*

# II. Sample composition of oriented samples

The exact composition of the PBLG-based weakly oriented samples used in this work is detailed in **Tables S1 and S2**

**Table S1.** Exact mesophase (PBLG/CHCl_3_) composition of (rac)-FLX samples

| **m_analyte_**  **(*rac*.)**  (mg) | **m_PBLG_**  **(DP = 641)**  (mg) | **m_solvant_** ^[a]^  (mg) | **[FLX]**  (mmol/l) | **m_poly_/m_tot_**  (%) |
| --- | --- | --- | --- | --- |
| 0.4 | 101.5 | 600.0 | 2.89 | 14.3 |
| 1.1 | 102.3 | 600.4 | 7.95 | 14.6 |
| 3.3 | 100.7 | 600.4 | 23.84 | 14.4 |
| 5.0 | 100.4 | 617.4 | 35.13 | 14.0 |
| 10.1 | 100.1 | 606.7 | 72.21 | 14.2 |
| 15.0 | 103.1 | 601.2 | 108.23 | 14.6 |
| 20.2 | 101.0 | 604.3 | 145.0 | 14.3 |
| 30.3 | 99.5 | 607.6 | 216.32 | 14.1 |

^[a]^ m_CHCl3 +_ m_analyte_

The sample containing 0.4 mg of FLX (last entry of **Table S1**) was prepared by dilution in order to minimize weighting errors in the case of low FLX mass weighting. An initial solution was prepared by mixing an accurate mass of (*rac)*-FLX (10.0 mg) with
1000 mg of CHCl_3_. A mass of solution was then sampled (40 mg) and CHCl_3_ was added until 600 mg of sample mass was reached. This solution was finally inserted into the NMR tube containing the polymer.

**Table S2.** Exact mesophase (PBLG/CHCl_3_) composition with enantiomeric excess of Fluoxetine samples

| ***ee* (*S*)**  (%) | **m_analyte_**  **(*rac*.)**  (mg) | **m_analyte_**  **(*S*)**  (mg) | **m_analyte_**  **(*tot*.)**  (mg) | **m_PBLG_**  **(DP = 641)**  (mg) | **m_solvent_** ^[a]^  (mg) | **m_pblg_/m_tot_**  (%) |
| --- | --- | --- | --- | --- | --- | --- |
| 0.0 | 10.1 | 0 | 10.1 | 100.1 | 606.7 | 14.2 |
| 8.9 $\pm$ 1.14 | 16.3 | 1.6 | 17.9 | 99.9 | 605.7 | 14.2 |
| 29.9 $\pm$ 1.14 | 7.2 | 3.0 | 10.2 | 100.0 | 602.5 | 14.2 |

^[a]^m_CHCl3 +_ m_analyte_

The scalemic sample (*ee* = 29.9%) of 10.2 mg (m_analyte_(tot.)) of FLX was prepared by dilution in order to minimize the theoretical enantiomeric excess uncertainty. An initial solution was prepared by mixing 14.3 mg of *(rac*)-FLX with 6.1 mg of (*S*)-FLX and 1200 mg of CHCl_3_. A mass of solution was sampled (602.5 mg) and added into the NMR tube with PBLG previously weighted.

The expected enantiomeric excess (*ee*_Theo_) was derived with the equation below:

$$ee(S)=\frac{m\left( S \right)-m(R)}{m\left( S \right)+m(R)}=\frac{m(S)}{m\left( S \right)+m(rac)}$$

The analytes were weighted with a balance (AB204 METTLER TOLEDO) claiming a standard deviation of 0.1 mg. Hence, weighing included an uncertainty $u$of $\pm$ 0.2 mg for a 95% confidence interval. These uncertainties are propagated as below to provide the total uncertainty of the prepared enantiomeric excess: *ee* (*S*) $\pm$ $U$:

#

$$\boldsymbol{U}\boldsymbol{=}\boldsymbol{ee}_{\boldsymbol{Theo}}\left( \boldsymbol{S} \right)\sqrt{\left( \frac{\boldsymbol{u}}{\boldsymbol{m(S)}} \right)^{\boldsymbol{2}}\boldsymbol{+}\frac{\boldsymbol{2}\boldsymbol{u}^{\boldsymbol{2}}}{{\boldsymbol{(m}\left( \boldsymbol{S} \right)\boldsymbol{+m(rac))}}^{\boldsymbol{2}}}}$$

# III. Investigation of Fluoxetine-PBLG contacts

Exploring the interaction array between Fluoxetine molecules and the PBLG side-chains can reveal valuable insights on the chiral recognition process. ^1^H saturation transfer difference (STD ^1^H NMR) experiments have been performed with that respect, a well-established method for studying of ligand-protein interactions [41]. This might provide some insights on the FLX moieties in contact with PBLG polymer (epitope mapping) by tracking the magnetization transfer (intermolecular nOe) between PBLG side-chains and the interacting FLX molecules. STD ^1^H NMR has been carried out in isotropic conditions with FLX samples (5 mg) containing a small amount of PBLG (10 mg in about 600 μL of CDCl_3_) since the anisotropic ^1^H NMR spectrum of FLX is not exploitable for STD experiments due to ubiquitous long-range residual ^1^H-^1^H dipolar couplings.

The STD ^1^H NMR experiments are conducted in the following conditions: a total number of scans of 256, an irradiation of PBLG via selective 40-ms Gaussian-shaped pulses during 2 s. Two irradiation frequencies: 6.0 and 4.9 ppm enable the saturation of the aromatic sites and at the vicinity of the carboxyl moiety of PBLG, respectively, without perturbation of FLX signals. A 80-ms *T*_2_-relaxation filter (CPMG) has been added prior the signal detection to remove the spectral pattern of the polymer overlapping the signals of interest. This relaxation filter also cancels the signal from the ammonium hydrogen of FLX. FID recorded in absence and presence of PBLG saturation are identically processed (manual phase correction, automatic polynomial baseline correction) and subtracted via the Bruker routine program “stdsplit” to get the STD spectrum. STD factors are calculated in percentage by comparing the STD signal intensities $I_{sat}$ *versus* those ($I_{0}$) of the reference spectrum (*i.e.*, without saturation):

STD factor (%) = $\frac{I_{0}-I_{sat}}{I_{0}}\times100$

The contact mapping is then (generally) given as a histogram with relative STD factors, *i.e.*, normalization by the highest STD factor.

A first STD spectrum recorded on a (*S*)-FLX sample after polymer irradiation at 6.0 ppm (aromatic part of PBLG, **Fig. S2A**), shows significant STD factors (cumulated sum of 48%), evidencing the presence of a non-negligible FLX-PBLG interaction. The relative STD histogram in **Fig. S2B** points out the high proximity of the stereogenic center (H6) of FLX to the PBLG side-chains. The aromatic hydrogens of the solute (H1-H5) are also closed, but at a lesser extent, in contrast to those of the alkyl part (H7, H8, CH_3_) providing rather poor STD factors.

To get further insights on the arrangement of FLX in contact with PBLG, we have conducted DiffErential Epitope (DEEP) mappings (DEEP-STD NMR) as proposed by the Angulo’s research group [42]. This is a method of choice to compare STD results from different experimental conditions, such as a change in irradiation frequency (6.0 *versus* 4.9 ppm, corresponding to different regions of the PBLG side chains). From DEEP-STD based on two different STD experiments (irradiation at 6.0 ppm as “exp1” and irradiation at 4.9 ppm as “exp2”), ΔSTD factors can be computed as below to assess the significance of changes in STD factors between the two experimental conditions [42]:

$\Delta$STD ($i$) = $\frac{{STD}_{\left( \exp1 \right)}(i)}{{STD}_{(exp2)}(i)}-\frac{1}{n}\sum_{i}^{n} \frac{{STD}_{\left( \exp1 \right)}(i)}{{STD}_{(exp2)}(i)}$

Where $n$ is the number of considered STD factors. |ΔSTD| > 0.4 is a threshold found in literature to distinguish significant and non-significant ΔSTD [43].

Several ΔSTD factors higher than 0.4 (in absolute values) are measured, confirming changes in the FLX epitope mapping under changes in the irradiation region of the polypeptide. In **Fig. S2C**, positive ΔSTD values reveal a higher proximity of FLX hydrogens to the PBLG terminal phenyl whereas negative ΔSTD suggests a closer contact with the PBLG ester group. The results underline here a preferential contact of FLX aromatic rings (H1-H5) with the phenyl groups of PBLG while most of the FLX aliphatic hydrogens (H7, H8, CH_3_) are closer to the ester function of the polymer. The insignificant ΔSTD value of H6 (< 0.4) reveals that the STD factor related to the asymmetric center is not disturbed by the shift in the irradiation frequency of PBLG from 6.0 to 4.9 ppm. This suggests a location of H6 at a similar distance from the ester and the phenyl group of the side chain.

Based on these results, we can outline an arrangement of FLX in contact of PBLG, in which the FLX aromatic groups interact with the PBLG terminal phenyl (presumably by π-π interactions) while the alkyl moiety of FLX is located at the vicinity of the carbonyl part of the polymer with an asymmetric center at a similar distance from the PBLG ester and the aromatic groups.

These results obtained on an enantiopur (*S*)-FLX sample are then compared to those collected on a racemic FLX sample analyzed in identical conditions to track potential discrepancies in interaction between the polypeptide and the two enantiomers. While the STD data are not strictly superimposable, the histograms in **Fig. S2B** and **S2B** show the same trend in relative STD and ΔSTD values. This suggests a similar arrangement of FLX enantiomers in contact with the PBLG side-chains. In order to underline the slight differences between enantiomers, the DEEP-STD approach is now applied on the (*S*)-FLX sample as “exp1” and on the (*rac*)-FLX sample as “exp2” (**Fig. S2D**). The low ΔSTD values (mostly < 0.4) of this histogram confirms the similarity of the transient complexes formed between PBLG and FLX enantiomers. Nonetheless, one may note a H8 and a methyl group slightly closer to PBLG terminal phenyl in the case of the (*S*)-enantiomer.

C

D

B

A

***Fig. S2:*** *(A) Structure of Fluoxetine (FLX) HCl along with atom numbering (top) and PBLG (bottom) with corresponding regions for 6.0 and 4.9 ppm irradiation. (B) Relative STD histograms (irradiation at 6.0 ppm during 2 s) for (S)-FLX and (rac)-FLX samples containing 5 mg of analyte and 10 mg of PBLG in CDCl_3_ (isotropic samples). (C) DEEP-STD histograms investigating the influence of the irradiation frequency (6.0 versus 4.9 ppm). (D) DEEP-STD histogram (irradiation at 6.0 ppm during 2 s) to track potential difference of epitope mapping between (S)-FLX versus (rac)-FLX samples.*

# IV. Longitudinal relaxation behavior of Fluoxetine

^19^F inversion-recovery NMR experiments with proton decoupling (Bruker pulse-program “t1irig”) were performed to measure the longitudinal relaxation times (*T*_1_) of the (*R*) and (*S*)-enantiomers. 16 increments with variable inversion delays were recorded with 16 scans per increment. The repetition time was fixed to 10.5 s to ensure full equilibrium magnetization before successive scans for a total duration of 48 min. The *T*_1_ values were extracted by non-linear fitting with a monoexponential function. This data treatment was carried out with the Dynamics module of Topspin software (Bruker).

A


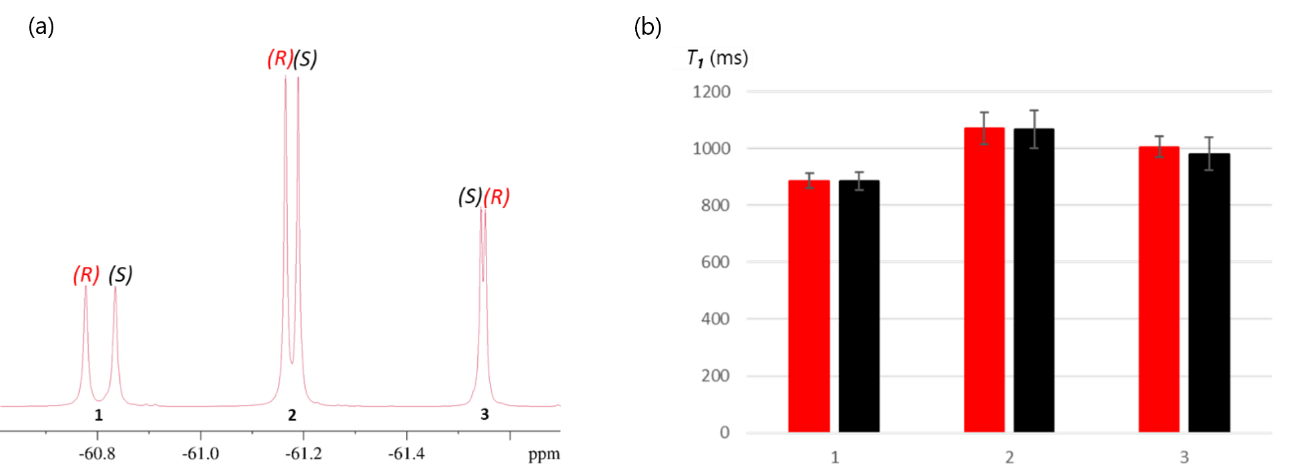


B

***Fig. S3:*** *(A) (282.4 MHz) ^19^F-{^1^H} NMR spectrum of (rac)-FLX (20 mg) dissolved in PBLG/CHCl_3_ at 300 K*. *(B) T_1_ relaxation times (ms) measured peak by peak for the two triplets. Error bars represent the 95% confidence interval determined as two times the standard deviation.*

# V. Spatial uniformity and stability of the lyotropic mesophases

The spatial uniformity and the stability of the lyotropic liquid crystals is crucial for the quality of the anisotropic NMR spectra. This is even more important to be studied here as FLX is added as a hydrochloride salt which may destabilize the helical structure of PBLG. This was investigated through ^19^F Z-imaging NMR experiments carried out with the pulse sequence below (**Fig. S4**). Note that the Z-axis matches with the direction of the NMR tube in the magnetic field.

C

B

A

**Fig. S4:** ^19^F Z-imaging NMR experiments on PBLG-based liquid crystals. (A) Schematic view of the NMR probe sensitive length (LS) along with the NMR tube. A train of spatially-selective experiments was performed with a slice thickness of $\Delta z$ thanks to the pulse-sequence shown in (B) where a shaped-pulse (qsneeze) was applied, together with a pulse-field-gradient G_S_. (C) An illustrative example of a pseudo-2D (Z, δ^19^F) showing the spatial dependence of the spectral data.

The spatial uniformity of the mesophase was investigated with various homogenization procedures. First, in the absence of any homogenization, the liquid-crystalline phase was not suitable for enantiomeric resolution, even after 15 days of equilibration (**Fig. S5**). Then, in case of manual homogenization, the situation is significantly better where a uniform mesophase was yielded three days after the sample preparation (**Fig. S6**). Finally, under successive centrifugation (ten times 30 s at 500 rpm), the liquid-crystalline phase was sufficiently uniform to address enantiomeric analysis as soon as the day of sample preparation (*i.e.*, D0) (**Fig. S7**). In addition, it is worth mentioning that the mesophase remains stable until at least 11 days, despite the presence of a HCl salt. This uniformity is robust again changes in experimental parameter (*e.g*., temperature and solute concentration) as seen in **Fig. S8**.

**Fig. S5:** Z-imaging ^19^F NMR experiments performed on a PBLG/CHCl_3_ liquid-crystalline phase with 108 mmol/L of FLX hydrochloride at different days DX, and in absence of any homogenization procedure. D0 corresponds to the day of sample preparation.

**Fig. S6:** Z-imaging ^19^F NMR experiments performed on a PBLG/CHCl_3_ liquid-crystalline phase with 108 mmol/L of FLX hydrochloride at different days DX, and under manual homogenization. D0 corresponds to the day of sample preparation.

**Fig. S7:** Z-imaging ^19^F NMR experiments performed on a PBLG/CHCl_3_ liquid-crystalline phase with 108 mmol/L of FLX hydrochloride at different days DX, and under several centrifugation cycles (ten times 30 s at 500 rpm). D0 corresponds to the day of sample preparation.

B

A

**Fig. S8**: Z-imaging ^19^F NMR experiments performed on a PBLG/CHCl_3_ liquid-crystalline phase with 5.0 mg of FLX hydrochloride (36 mmol/L) recorded at 27°C (A) and 37°C (B). The 1D spectra (in blue) displayed in F_2_ projections correspond to the ^19^F-{^1^H} 1D spectra recorded on the entire sensitive volume of the NMR probe coil.

# VI. NMR spectral data of (*rac*)-Fluoxetine dissolved in PBLG/CHCl_3_ at various concentrations and temperatures

All the data below were recorded from PBLG-based liquid crystals prepared with 14 wt% in polymer (see the sample compositions in **Table S1**). Only the FLX concentration and the sample temperature vary in this series of NMR experiments. The measurements of the FLX anisotropic parameters are exemplified in **Fig. S2** and **S3**.

**Table S3.** Variation of anisotropic parameters for a 0.4 mg of (*rac*)-Fluoxetine dissolved in PBLG/CHCl_3_ at *T* = 300 K

| **T**  (K) | $\left\vert\boldsymbol{T}_{\mathbf{FF}} \right\vert\boldsymbol{(R)}$  (Hz) | $\left\vert\boldsymbol{T}_{\mathbf{FF}} \right\vert\boldsymbol{(S)}$  (Hz) | $\left\vert\left\vert\boldsymbol{T}_{\mathbf{FF}} \right\vert\left( \boldsymbol{R} \right)\boldsymbol{-}\left\vert\boldsymbol{T}_{\mathbf{FF}} \right\vert\left( \boldsymbol{S} \right) \right\vert$  (Hz) | **^19^F** $\left\vert\boldsymbol{\Delta\Delta\delta}\left( \boldsymbol{R,S} \right) \right\vert$  (ppm) |
| --- | --- | --- | --- | --- |
| 297 | 55.8 | 16.1 | 39.7 | 0.187 |
| 300 | 52.1 | 23.1 | 29.1 | 0.196 |
| 305 | 45.4 | 37.0 | 8.4 | 0.215 |
| 310 | 38.3 | 52.4 | 14.1 | 0.236 |
| 315 | 30.7 | 67.7 | 37.0 | 0.257 |

**Table S4.** Variation of anisotropic parameters for a 1.1 mg of (*rac*)-Fluoxetine dissolved in PBLG/CHCl_3_ at *T* = 300 K

| **T**  (K) | $\left\vert\boldsymbol{T}_{\mathbf{CH}} \mathbf{(}\mathbf{CHCl}_{\mathbf{3}}\mathbf{)} \right\vert$  (Hz) | $\left\vert\boldsymbol{T}_{\mathbf{FF}} \right\vert\boldsymbol{(R)}$  (Hz) | $\left\vert\boldsymbol{T}_{\mathbf{FF}} \right\vert\boldsymbol{(S)}$  (Hz) | $\left\vert\left\vert\boldsymbol{T}_{\mathbf{FF}} \right\vert\left( \boldsymbol{R} \right)\boldsymbol{-}\left\vert\boldsymbol{T}_{\mathbf{FF}} \right\vert\left( \boldsymbol{S} \right) \right\vert$  (Hz) | **^19^F** $\left\vert\boldsymbol{\Delta\Delta\delta}\left( \boldsymbol{R,S} \right) \right\vert$  (ppm) |
| --- | --- | --- | --- | --- | --- |
| 297 | 313.9 | 75.6 | 22.9 | 52.7 | 0.138 |
| 300 | 311.5 | 73.5 | 20.1 | 53.4 | 0.140 |
| 305 | 307.5 | 69.6 | 14.9 | 54.7 | 0.144 |
| 310 | 303.6 | 65.7 | 9.1 | 56.6 | 0.148 |
| 315 | 300.0 | 61.4 | 2.9 | 58.5 | 0.153 |

**Table S5.** Variation of anisotropic NMR observables for a 3.3 mg of (*rac*)-Fluoxetine Fluoxetine dissolved in PBLG/CHCl_3_ at *T* = 300 K

| **T**  (K) | $\left\vert\boldsymbol{T}_{\mathbf{CH}} \mathbf{(}\mathbf{CHCl}_{\mathbf{3}}\mathbf{)} \right\vert$  (Hz) | $\left\vert\boldsymbol{T}_{\mathbf{FF}} \right\vert\boldsymbol{(R)}$  (Hz) | $\left\vert\boldsymbol{T}_{\mathbf{FF}} \right\vert\boldsymbol{(S)}$  (Hz) | $\left\vert\left\vert\boldsymbol{T}_{\mathbf{FF}} \right\vert\left( \boldsymbol{R} \right)\boldsymbol{-}\left\vert\boldsymbol{T}_{\mathbf{FF}} \right\vert\left( \boldsymbol{S} \right) \right\vert$  (Hz) | **^19^F** $\left\vert\boldsymbol{\Delta\Delta\delta}\left( \boldsymbol{R,S} \right) \right\vert$  (ppm) |
| --- | --- | --- | --- | --- | --- |
| 297 | 310.3 | 91.2 | 57.1 | 34.1 | 0.090 |
| 300 | 310.2 | 89.5 | 55.3 | 34.2 | 0.090 |
| 305 | 306.3 | 86.5 | 52.4 | 34.1 | 0.090 |
| 310 | 302.6 | 83.5 | 49.4 | 34.1 | 0.090 |
| 315 | 298.9 | 80.6 | 46.7 | 33.9 | 0.089 |

**Table S6**: Variation of anisotropic NMR observables for a 5.0 mg racemic sample of FLX dissolved in PBLG/CHCl_3_ at *T* = 300 K

| **T**  (K) | $\left\vert\boldsymbol{T}_{\mathbf{CH}} \mathbf{(}\mathbf{CHCl}_{\mathbf{3}}\mathbf{)} \right\vert$  (Hz) | $\left\vert\boldsymbol{T}_{\mathbf{FF}} \right\vert\boldsymbol{(R)}$  (Hz) | $\left\vert\boldsymbol{T}_{\mathbf{FF}} \right\vert\boldsymbol{(S)}$  (Hz) | $\left\vert\left\vert\boldsymbol{T}_{\mathbf{FF}} \right\vert\left( \boldsymbol{R} \right)\boldsymbol{-}\left\vert\boldsymbol{T}_{\mathbf{FF}} \right\vert\left( \boldsymbol{S} \right) \right\vert$  (Hz) | **^19^F** $\left\vert\boldsymbol{\Delta\Delta\delta}\left( \boldsymbol{R,S} \right) \right\vert$  (ppm) |
| --- | --- | --- | --- | --- | --- |
| 297 | 308.0 | 87.8 | 63.3 | 24.5 | 0.065 |
| 300 | 305.5 | 87.8 | 63.2 | 24.6 | 0.065 |
| 305 | 301.7 | 85.5 | 61.0 | 24.5 | 0.065 |
| 310 | 298.1 | 83.1 | 58.8 | 24.3 | 0.064 |
| 315 | 294.6 | 80.9 | 56.6 | 24.3 | 0.063 |

**Table S7.** Variation of anisotropic NMR observables for a 10.1 mg of (*rac*)-Fluoxetine in PBLG/CHCl_3_ at *T* = 300 K

| **T**  (K) | $\left\vert\boldsymbol{T}_{\mathbf{FF}} \right\vert\boldsymbol{(R)}$  (Hz) | $\left\vert\boldsymbol{T}_{\mathbf{FF}} \right\vert\boldsymbol{(S)}$  (Hz) | $\left\vert\left\vert\boldsymbol{T}_{\mathbf{FF}} \right\vert\left( \boldsymbol{R} \right)\boldsymbol{-}\left\vert\boldsymbol{T}_{\mathbf{FF}} \right\vert\left( \boldsymbol{S} \right) \right\vert$  (Hz) | **^19^F** $\left\vert\boldsymbol{\Delta\Delta\delta}\left( \boldsymbol{R,S} \right) \right\vert$  (ppm) |
| --- | --- | --- | --- | --- |
| 297 | 104.8 | 92.1 | 12.7 | 0.033 |
| 300 | 103.3 | 90.9 | 12.4 | 0.033 |
| 305 | 101.0 | 88.3 | 12.7 | 0.033 |
| 310 | 98.8 | 86.2 | 12.6 | 0.033 |
| 315 | 96.7 | 84.0 | 12.7 | 0.034 |

**Table S8.** Variation of anisotropic NMR observables for a 15.0 mg of (*rac*)-Fluoxetine dissolved in PBLG/CHCl_3_ at *T* = 300 K

| **T**  (K) | $\left\vert\boldsymbol{T}_{\mathbf{FF}} \right\vert\boldsymbol{(R)}$  (Hz) | $\left\vert\boldsymbol{T}_{\mathbf{FF}} \right\vert\boldsymbol{(S)}$  (Hz) | $\left\vert\left\vert\boldsymbol{T}_{\mathbf{FF}} \right\vert\left( \boldsymbol{R} \right)\boldsymbol{-}\left\vert\boldsymbol{T}_{\mathbf{FF}} \right\vert\left( \boldsymbol{S} \right) \right\vert$  (Hz) | **^19^F** $\left\vert\boldsymbol{\Delta\Delta\delta}\left( \boldsymbol{R,S} \right) \right\vert$  (ppm) |
| --- | --- | --- | --- | --- |
| 297 | 113.5 | 103.1 | 10.4 | 0.028 |
| 300 | 112.0 | 101.7 | 10.3 | 0.028 |
| 305 | 109.5 | 99.6 | 9.9 | 0.027 |
| 310 | 107.4 | 97.0 | 10.4 | 0.027 |
| 315 | 104.8 | 94.8 | 10.0 | 0.027 |

**Table S9.** Variation of anisotropic NMR observables for a 20.2 mg of (*rac*)-Fluoxetine in PBLG/CHCl_3_ at *T* = 300 K

| **T**  (K) | $\left\vert\boldsymbol{T}_{\mathbf{FF}} \right\vert\boldsymbol{(R)}$  (Hz) | $\left\vert\boldsymbol{T}_{\mathbf{FF}} \right\vert\boldsymbol{(S)}$  (Hz) | $\left\vert\left\vert\boldsymbol{T}_{\mathbf{FF}} \right\vert\left( \boldsymbol{R} \right)\boldsymbol{-}\left\vert\boldsymbol{T}_{\mathbf{FF}} \right\vert\left( \boldsymbol{S} \right) \right\vert$  (Hz) | **^19^F** $\left\vert\boldsymbol{\Delta\Delta\delta}\left( \boldsymbol{R,S} \right) \right\vert$  (ppm) |
| --- | --- | --- | --- | --- |
| 297 | 110.8 | 103.2 | 7.6 | 0.020 |
| 300 | 109.3 | 101.9 | 7.4 | 0.020 |
| 305 | 107.1 | 99.8 | 7.3 | 0.020 |
| 310 | 104.8 | 97.5 | 7.3 | 0.020 |
| 315 | 102.8 | 95.3 | 7.5 | 0.020 |

**Table S10.** Variation of anisotropic NMR observables for a 30.3 mg of (*rac*)-Fluoxetine dissolved in PBLG/CHCl_3_ at *T* = 300 K

| **T**  (K) | $\left\vert\boldsymbol{T}_{\mathbf{FF}} \right\vert\boldsymbol{(R)}$  (Hz) | $\left\vert\boldsymbol{T}_{\mathbf{FF}} \right\vert\boldsymbol{(S)}$  (Hz) | $\left\vert\left\vert\boldsymbol{T}_{\mathbf{FF}} \right\vert\left( \boldsymbol{R} \right)\boldsymbol{-}\left\vert\boldsymbol{T}_{\mathbf{FF}} \right\vert\left( \boldsymbol{S} \right) \right\vert$  (Hz) | **^19^F** $\left\vert\boldsymbol{\Delta\Delta\delta}\left( \boldsymbol{R,S} \right) \right\vert$  (ppm) |
| --- | --- | --- | --- | --- |
| 297 | 107.7 | 103.6 | 4.1 | 0.015 |
| 300 | 106.5 | 102.4 | 4.1 | 0.014 |
| 305 | 104.2 | 100.2 | 4.0 | 0.014 |
| 310 | 101.9 | 98.2 | 3.7 | 0.013 |
| 315 | 99.8 | 96.4 | 3.4 | 0.012 |

The orienting properties of the liquid crystals according to the concentration of hydrochloride Fluoxetine were monitored by the measurement of the total coupling *T*_CH_ of the co-solvent (CHCl_3_). This coupling constant remains stable with the FLX concentration, as depicted by a small relative standard deviation (0.76%). Moreover, the dispersion of *T*_CH_ values is random showing the absence of trend (**Fig. S9**).


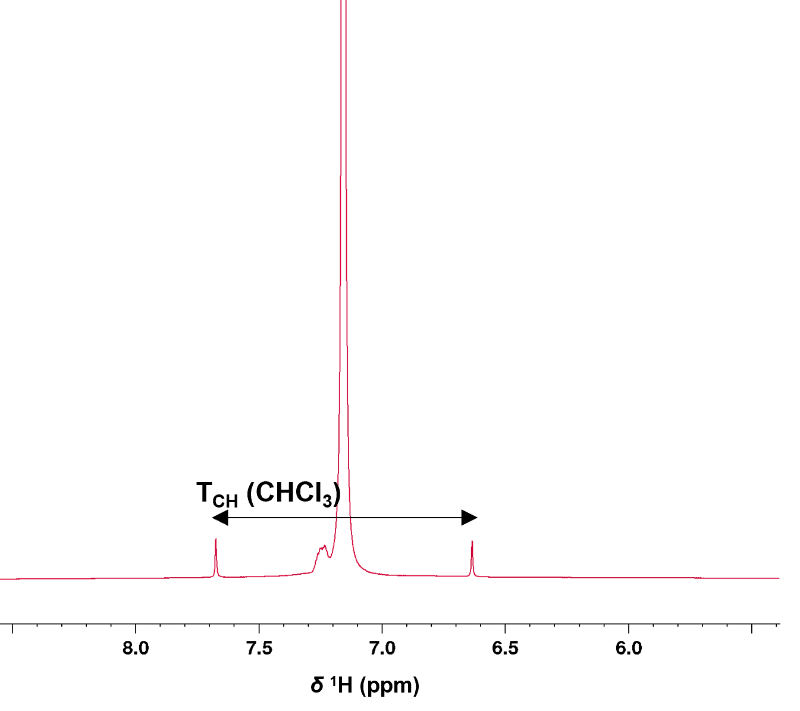


**Fig. S9:** Zoom of 300 MHz ^1^H NMR spectrum of (rac)-FLX dissolved in PBLG/CHCl_3_ and showing the ^13^C-satellites signals (0.5 %) used for the measurement of $\left| T_{CH} \right|$ of CHCl_3_.


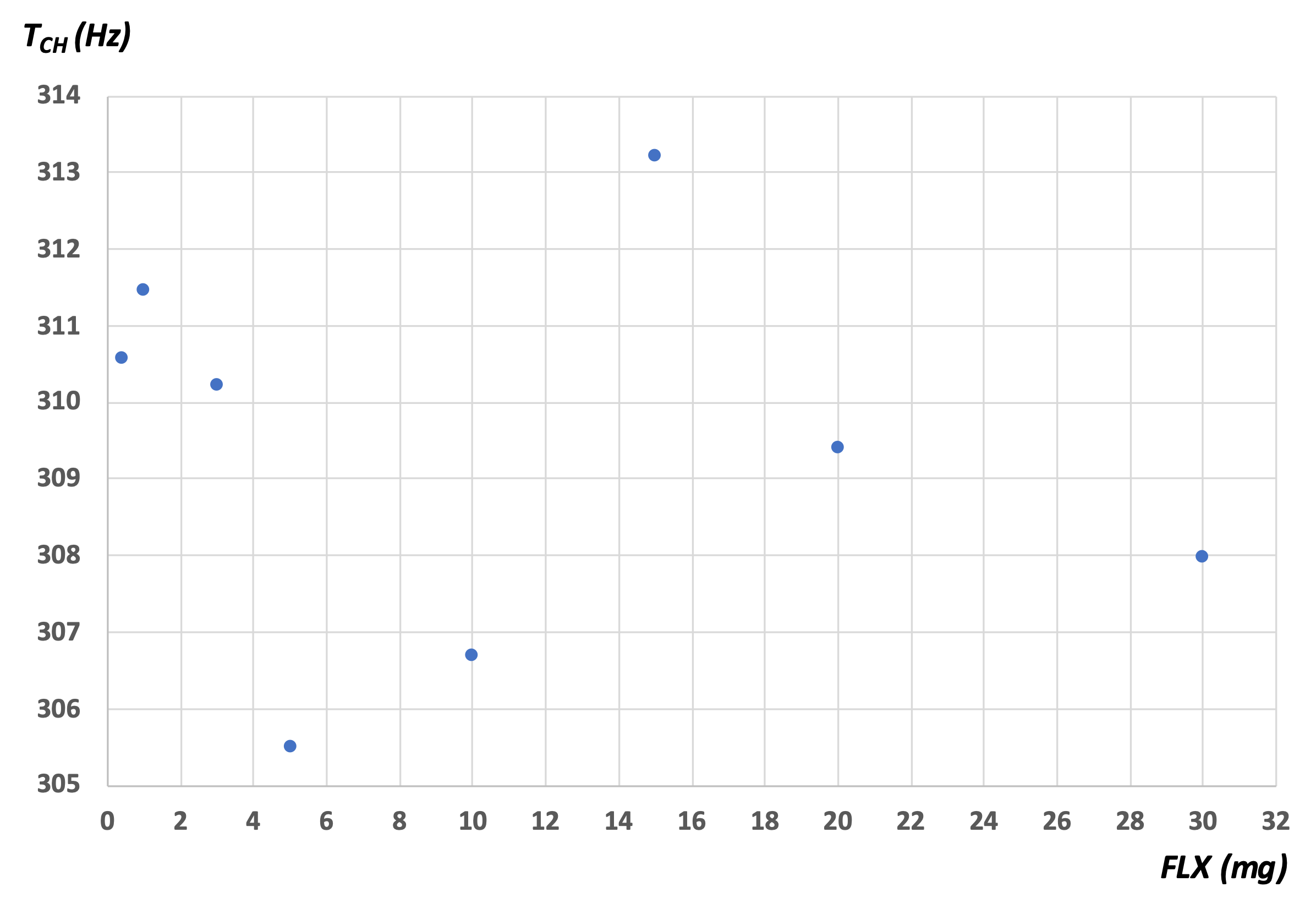


***Fig. S10:*** *Plot of* $\left| T_{\mathrm{CH}} \right|$ *values (Hz) of CHCl_3_ as a function of the mass of (rac)-FLX (mg) in PBLG/CHCl_3_.*

**Table S11.** *T*_CH_ values of CHCl_3_ as a function of the mass of FLX in PBLG/CHCl_3_

| FLX (mg) | 0.4 | 1 | 3 | 5 | 10 | 15 | 20 | 30 |
| --- | --- | --- | --- | --- | --- | --- | --- | --- |
| $\left\vert T_{CH} \right\vert$ (Hz) | 310.6 | 311.5 | 310.2 | 305.5 | 306.7 | 313.2 | 309.4 | 308.0 |

| Mean value (Hz) | SD (Hz) | RSD (%) |
| --- | --- | --- |
| 309.4 | 4.8 | 0.8 |
